# Imaging Fibrosis and Separating Collagens using Second Harmonic Generation and Phasor Approach to Fluorescence Lifetime Imaging

**DOI:** 10.1038/srep13378

**Published:** 2015-08-21

**Authors:** Suman Ranjit, Alexander Dvornikov, Milka Stakic, Suk-Hyun Hong, Moshe Levi, Ronald M. Evans, Enrico Gratton

**Affiliations:** 1 Laboratory for Fluorescence Dynamics, Department of Biomedical Engineering, University of California Irvine, California; 2 Division of Renal Diseases and Hypertension, University of Colorado at Denver, Aurora, Colorado; 3 Gene Expression Laboratory, Salk Institute for Biological Studies, La Jolla, CA

## Abstract

In this paper we have used second harmonic generation (SHG) and phasor approach to auto fluorescence lifetime imaging (FLIM) to obtain fingerprints of different collagens and then used these fingerprints to observe bone marrow fibrosis in the mouse femur. This is a label free approach towards fast automatable detection of fibrosis in tissue samples. FLIM has previously been used as a method of contrast in different tissues and in this paper phasor approach to FLIM is used to separate collagen I from collagen III, the markers of fibrosis, the largest groups of disorders that are often without any effective therapy. Often characterized by an increase in collagen content of the corresponding tissue, the samples are usually visualized by histochemical staining, which is pathologist dependent and cannot be automated.

Fibrotic diseases are responsible for organ death and often the only possible course of action is exchange with a healthy organ^1^. The various diseases associated with the fibrosis include liver cirrhosis, idiopathic pulmonary fibrosis, diabetic nephropathy, arteriosclerosis, scleroderma, rheumatoid arthritis and fibrosarcomas^1^,^2^,^3^,^4^,^5^,^6^,^7^. Fibrotic diseases are one of the largest groups of disorders without any effective therapy. They usually arise as the wound healing process fails to end after the normal wound healing response. During this wound healing process new tissues are synthesized and the proteins being produced include collagens and fibronectins. Failure to end this synthesis results in overproduction of fibril forming proteins and fibrosis^1^,^8^. Collagens are one of the major components of the extracellular matrix (ECM) in tissues and are the major components of fibrosis^7^,^8^. They are the most abundant proteins in the human body, consisting of almost 30% of the total protein mass. There are various different types of collagens that are present in mammalian systems, some fibrous and some non-fibrous. The fibrous collagens give rise to complicated fibril structures and are responsible for the tensile strength and fibrillar network. The non-fibrous collagens are responsible for various other biological functions including tissue flexibility^8^. It has also been shown that the ratio of collagen III to collagen I is important for diseases like dilated cardiomyopathy and fibrosis^3^,^5^,^9^. Collagen I is mostly heterotrimeric, non-centrosymmetric and the most abundant in the tissues. Collagen III is often co-distributed with collagen I. The major source of type IV collagen is basement membranes and type V collagen is usually present in a small amount at the core of the collagen I fibers^8^.

Collagens have been studied using various different techniques, including immunohistochemical staining of the excised tissue to determine the type of collagen, second harmonic generation (SHG) imaging, and HPLC combined with mass spectrometry^6^,^8^,^10^,^11^,^12^,^13^. Fluorescence imaging and fluorescence lifetime imaging (FLIM) have also been employed, although not for separating different fluorescence components^4^,^14^,^15^,^16^,^17^,^18^. These fluorescence techniques were used for the characterization of collagen and separating collagens from other tissue components, but not for separating different types of collagens. The main way to separate different collagens to date has been staining the excised tissues with the dye, picrosirius red^19^. This technique although widely employed, is pathologist dependent and cannot be automated. SHG have also been widely used to image collagen fibrils. The non-centrosymmetric structure of some collagen fibers give rise to SHG signals and can be used for imaging^11^. The caveat is that SHG cannot be used for either the non-fibrous or for the symmetric fibrous collagen samples^2^,^10^,^16^,^17^,^20^. Amongst the fibril forming collagens, collagen I and II result in the strongest SHG signals and collagen III, although fibrous, result in very weak SHG signals^10^. It has also been shown that in a gel formed from the mixture of collagen I and V, increasing the fraction of collagen V results in smaller fiber formation and fibers of smaller diameter. The collagen V usually forms the core of the fiber and is usually wrapped around by collagen I. Thus collagen V by itself does not give good SHG contrast^21^,^22^. Accordingly SHG being the most widely used technique for the label free imaging of collagens, cannot be used for the imaging of all types of collagens. HPLC followed by mass spectroscopy has also been employed for the separation of collagen signals and have been shown to separate signatures of collagen I through V. However, this technique is incapable of giving images and hence the localization of different collagens in different tissues cannot be visualized^8^.

Collagens are known to show autofluorescence when excited with single photon UV excitation or with a two photon excitation around 730 nm. The fibrous collagen I, when excited with 730 nm light in two-photon excitation scheme, shows auto-fluorescence at 450 nm to 600 nm wavelength range^17^,^20^. These fibers were determined to have bi-exponential fluorescence lifetime decay with 39% amplitude of 0.29 ns and 61% amplitude of 1.68 ns^15^. The fluorescence properties of collagens, mostly collagen I in solution, including one and two photon excitation spectra, absorption spectra, excitation dependent emission spectra, and fluorescence lifetime have been known in literature^23^,^24^. However, the properties of the fibrous form of collagen I are very different compared to the properties in solution^15^,^20^. Even though this vast amount of knowledge about fluorescence of collagens has been known, it has never been employed to separate out the signatures arising from different collagens in their native like structures.

In this paper, first we present FLIM as a technique to separate different collagen signatures (collagen I through V) in the pre-formed gels^14^,^15^,^25^. The phasor approach to lifetime, which offers a fit free method of separating pixels having different fluorescence lifetimes, is used for the analysis of the FLIM images^26^,^27^,^28^,^29^,^30^,^31^. In this approach, populations having similar lifetimes can be selected in the phasor plot and the fluorescence image is painted accordingly. This approach was first used to find the signature positions in the phasor plot of the different collagen autofluorescence from the individual gels and then further used to separate collagen I and III in the SMRT^mRID^ mouse femur. The SMRT^mRID^ mouse produces spontaneous myelofibrosis, a progressive bone marrow fibrosis and results in increasing collagen I and collagen III in the bone marrow samples^32^. Combined with the SHG, FLIM and the phasor approach represent a new method of separating different collagens in an image with a label free approach and gives the possibility to use it as a diagnostic tool for fibrosis.

## Results and Discussion

### Pure collagen gels

**Figure 1:**
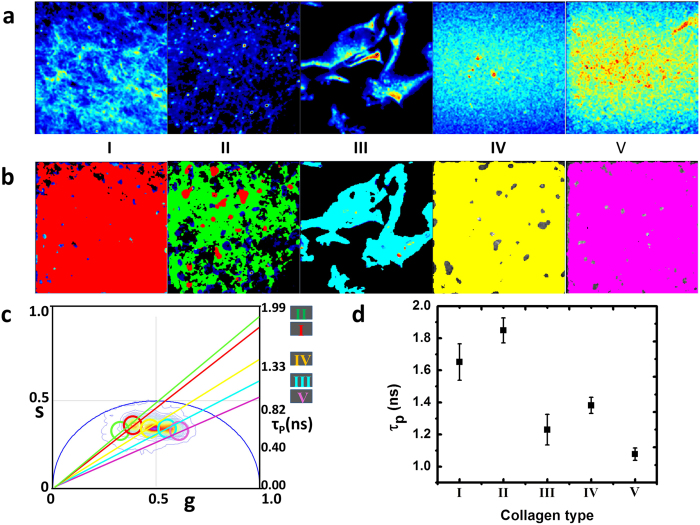
Separation of collagens based on the fluorescence lifetime using the clusters in the phasor plot. Figure 1a, from left to right, shows the fluorescence intensity signals originating from gels of collagen I to V. Figure 1b shows the same intensity image masked with the cursor color chosen in the phasor plot (Fig.1c). Red, green, cyan, yellow and magenta colors were chosen to select the phasor clusters in Fig. 1c and the intensity images were painted correspondingly. Figure 1d shows the calculated phase lifetimes and shows the separation in phasor plot (Fig. 1c) is more significant. The field of view in these images is 38 μm.

The objective of this paper was to identify locations in the phasor plot that can be used to separate different collagen types in an image. Collagen gels consisting of purified collagen I through V were prepared and imaged with a 32 μs pixel dwell time for 20 repeat images. The images were acquired with a 38 μm field of view and a resolution of 256 × 256 pixels. Each individual fluorescence image was first corrected for the background. The position of the phasor points in the phasor plot originating from that particular image were selected with a colored cursor and the image was painted accordingly. Figure 1a shows the intensity images after background correction (from left to right are collagen I through V). The corresponding FLIM images (Fig. 1b) were colored according to the chosen cursors in Fig. 1c. In the phasor plot (Fig. 1c) each type of collagen forms a different cluster of phasor points. Each cluster is indicated by a circular colored cursor. The points in the images are colored according to the cluster they belong. The red, green, cyan, yellow and magenta represent the selected phasor points for collagen I, II, III, IV and V, respectively. Figure 1b, showing the intensity image painted with the chosen cursor colors, proves that the clusters in the phasor plot are completely separate and can be used to identify the type of collagen.

Each point in the phasor plot can be associated with a phase angle ϕ and a distance from the origin. We define the lifetime obtained from the phase of each point in the phasor plot as
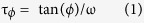
where,
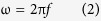
and f is the repetition frequency of the laser (80 MHz). The average phase lifetimes of the collagens are shown in Fig. 1d. The average phase lifetime for the collagens is around 1.5 ns which agree with previously known values^33^. This figure also underlines the importance of the phasor approach to FLIM analysis. All of the five collagens measured using the phase lifetime have lifetime between 1 ns to 2 ns, and cannot be easily separated. However they are clearly separated in the phasor plot of Fig. 1c.

**Figure 2:**
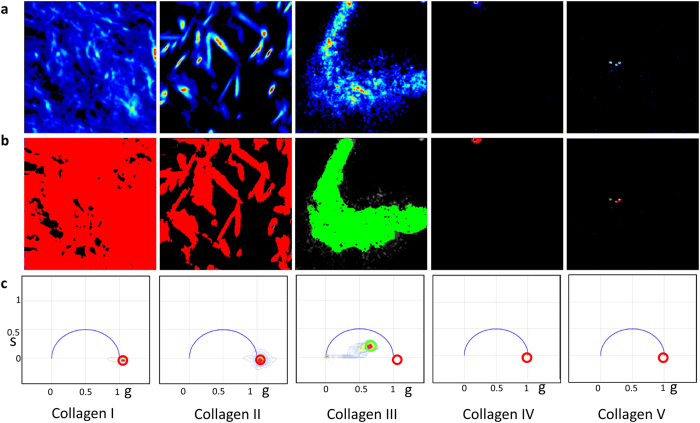
Signals in the SHG channel for gels of collagen I to V. (Fig. 2a) SHG intensity image of collagen I to V (left to right). (Fig. 2b) SHG intensity images overlapped with the color mask chosen in the phasor plots (Fig. 2c). Red cursor was used to select the phasor points of zero lifetime (SHG) and the green cursor was used to select the fluorescence phasor points (non-zero lifetime). It is evident that the signal in the SHG channel for the collagen III (Fig. 2a) can be identified with fluorescence since the position in the phasor plot is not at the (1,0) position.

The other important observation is that the relative intensity of SHG and fluorescence are dependent on the type of collagens. Collagen I and II give very strong second harmonic signals. Collagen IV and V do not produce SHG. Collagen III has a weak contribution in the SHG channel, but this signal in our instrument is due to leakage of the very strong fluorescence signal through the filters used for the separation of SHG signals, as collagen III is responsible for the strongest fluorescence amongst the collagens under study. Figure 2a shows the SHG intensity images acquired in the same field of view as that of the fluorescence images of Fig. 1a. The phasor points originating from SHG images appear at the coordinate of s = 0 and g = 1, as the lifetime of SHG is basically zero. Figure 2c shows the phasor plots arising from each of the five collagen SHG images. The phasor plot for collagen III has a non-zero lifetime and is similar to the fluorescence lifetime of collagen III, thus signifying that the origin of the signal for collagen III is not SHG and is actually fluorescence. After selection of the different populations of the phasor plot (Fig. 2c) using colored cursors, the image for collagen I and II the images were masked with red (SHG mask) and the collagen III image was masked with green, the mask for non-zero lifetime. On the contrary, collagens IV and V do not produce any SHG signal. Collagen IV has a non-fibrous structure and hence does not produce second harmonic signals. Collagen V is known to be fibrillar, but only in the presence of collagen I. In a gel formed from the mixture of collagen I and V, increasing fraction of collagen V results in decrease of fibrillar structure and also a decrease in the fibril diameter. An increase of 20% of collagen V in the mixture of collagen I and V decreases the fibril structures by 40%. Thus a gel formed by only collagen V does not produce fibrillar structure and SHG signals^21^.

The relative intensities obtained in the fluorescence and SHG images of the different collagens acquired under same laser power indicate the possibility of separating collagens based on the ratio of the SHG and fluorescence signals. Collagen IV and V have almost no SHG signals, thus they can only be separated by the FLIM analysis. For the other three collagens the ratio of the intensities of SHG and fluorescence signals acquired in the same field of view were calculated and is shown in Supplementary Figure S1. The Y axis in this figure is in semi logarithmic scale and thus the very large difference shown in the SHG to fluorescence intensity ratios of these different collagens represent the large separation of collagen type based on this criteria.

### Collagen mixtures in gels

**Figure 3:**
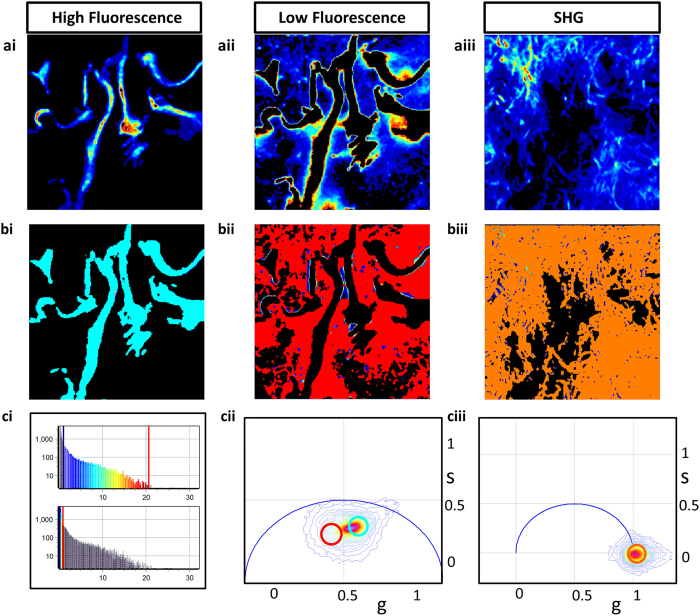
Fluorescence and SHG signals of the gel prepared from the mixture of collagen I and III. The fluorescence image was selected either for the high intensity (Fig. 3ai) using the top histogram (Fig. 3ci) or for the low intensity (Fig. 3aii) using the bottom histogram in (Fig. 3ci). The fluorescence intensity images were masked using the cursor colors in the phasor plot (Fig. 3cii) and colored accordingly to show the prominently collagen III rich region (Fig. 3bi) and collagen I rich region (Fig. 3bii). The SHG intensity image, phasor masked image and the corresponding phasor plot for SHG generation is shown in Fig. 3aiii–3ciii, respectively.

Collagen I and III are known to coexist in tissues. Both the ratio of the two collagens and the total amount of them has been shown to indicate the extent of different fibrotic diseases. As mentioned earlier, collagen I results in stiffness and tensile strength and large amount of collagen III results in greater elasticity. Thus a change in the relative ratios of these two collagens can determine the behavior of the extra cellular matrix and was shown to be an important factor in cardiac myopathy^3^,^4^,^5^. Therefore separating the signals of collagen I and III becomes important for diagnostic purposes. To distinguish if the phasor approach to FLIM can separate the collagen I and III signals, a mixed gel was formed from a 3:1 mixture of these two collagens and then SHG and FLIM images were acquired. Figure 3ai,ii show the fluorescence images selected for regions of high intensity and the low intensity, respectively. This selection was done based on the histogram in Fig. 3ci, where the top shows the selection for the Fig. 3ai and the bottom shows the selection for the Fig. 3aii. Collagen III is much more fluorescent than collagen I and thus to observe collagen I, the lower fluorescence intensity must be selected. Figure 3bii,bi show the masked image of the intensity overlapped with the phasor color in Fig. 3cii. In Fig. 3bi, most of the image is colored cyan and in Fig. 3bii most of the image is colored red, the cursor colors (Fig. 3ci) for the FLIM signature of collagen III and I, respectively. Figure 3aiii–ciii show the SHG intensity image, phasor masked image and the phasor plot of the second harmonic generation, respectively. The lifetime of SHG is zero and thus the phasor points appears at s = 0, g = 1 (Fig. 3ciii). A comparison between Fig. 3bii,biii shows that most of the fiber structures in the SHG image can also be separated by the red fluorescence mask in Fig. 3biii. Collagen I has a very strong SHG signal. In the mixture, the bleed through of collagen III fluorescence in the SHG channel has a much lower intensity than the SHG signal of collagen I and is actually very close to the background. Thus after background correction, the phasor points originating from the bleed through disappears from the phasor plot (Fig. 3ciii). It is also evident that the bright image in Fig. 3ai does not give rise to the signals in the SHG channels and only the dim fluorescent spots, i.e. the ones from collagen I coexist in both SHG and fluorescence images. This proves that at least in gels, collagen I and III can be separated based on lifetime.

### Fibrosis in biological samples

**Figure 4:**
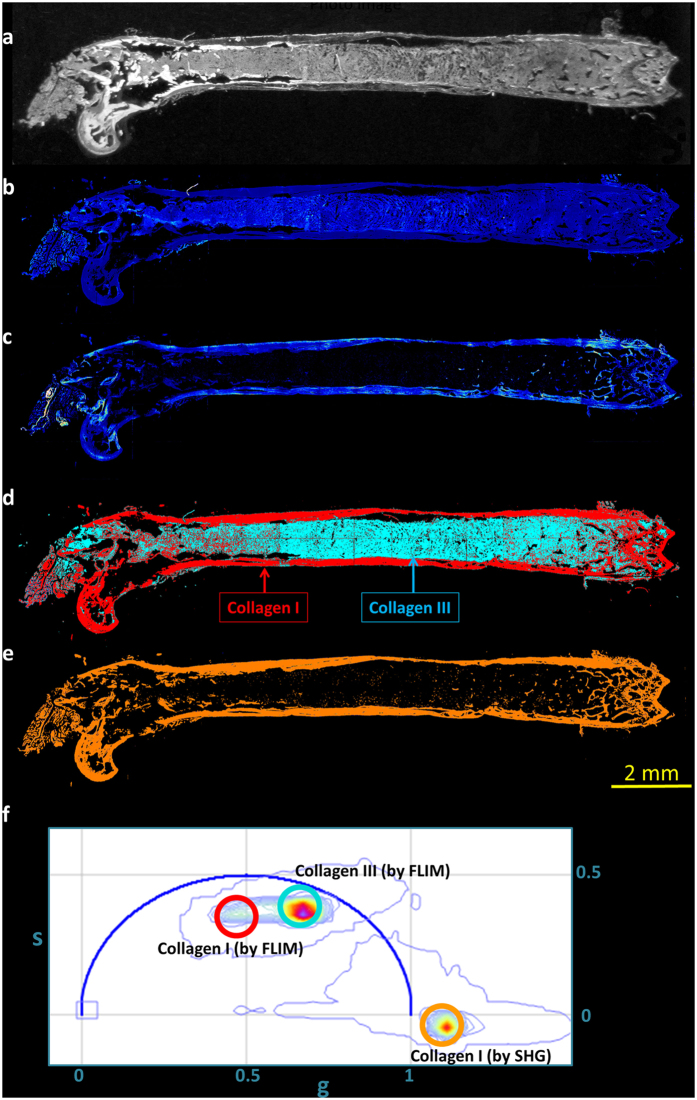
Separation of collagen I and collagen III in mouse femur bones. Picture of the bone, two photon auto-fluorescence intensity image and the SHG images are shown in Fig. 4a–c, respectively. The FLIM and SHG phasor masked images are shown in Fig. 4d,e where the masking color indicates the cursor color of the phasor plot (Fig. 4f). Most of the parts chosen by cyan (Fig. 4d) are not present in Fig. 4e and mostly the red part of the masked image correlates with the SHG image.

A mouse model that spontaneously develops myelofibrosis is the SMRT^mRID^ mouse. In these mice, two receptor interaction domains of epigenetic repressor silencing mediator of retinoid and thyroid hormone receptors are targeted and these mice develops spontaneous myelofibrosis, characterized by the bone marrow fibrosis and increasing collagen content in the bone marrow^32^. The FLIM and SHG measurements were further extended to study these mouse femur slices, obtained from Dr. Ronald Evans’ lab at Salk Institute, San Diego, CA. Each individual image was taken with 925 μm field of view and with 256 × 256 pixels. Both FLIM and SHG images were then tiled and are shown in Fig. 4b,c, respectively. Figure 4a shows the image of the bone taken with a camera. Red, cyan and orange colored cursors were chosen in the phasor plot (Fig. 4f) to select collagen I fluorescence, collagen III fluorescence and the SHG signals, respectively. Fig. 4d,e represent the phasor masked image of the corresponding intensity images, Fig. 4b,c. A comparison between the masked FLIM (Fig. 4d) and masked SHG (Fig. 4e) shows that the part of the image covered in SHG is mostly covered by red in the fluorescence image, red being the cursor used for collagen I FLIM signature, signifying the inability of collagen III to produce SHG. This result shows that in the mouse femur tissue, collagen I and III can be separated by the FLIM. The strong correlation between the pixels measured by SHG and the red mask (the mask for collagen I) in the FLIM images shows that collagen I can be identified by both SHG and FLIM (Supplementary Fig. S2).

The phasor approach to separate collagens using FLIM imaging was further extended to study myelofibrosis in the bone marrows. The two components of the bones, osteoblasts and osteoclasts maintain a balance where osteoblasts absorb the bone matrix and osteoclasts regenerate the matrix continuously. In case of idiopathic myelofibrosis the bone marrows gets occupied by fibrotic tissues, e.g. collagens, and changes the microenvironment of the bone marrow. Most of the treatments available for the myelofibrosis are supportive and the one main treatment is the significantly risky allogenic stem cell transplantation^32^.

**Figure 5:**
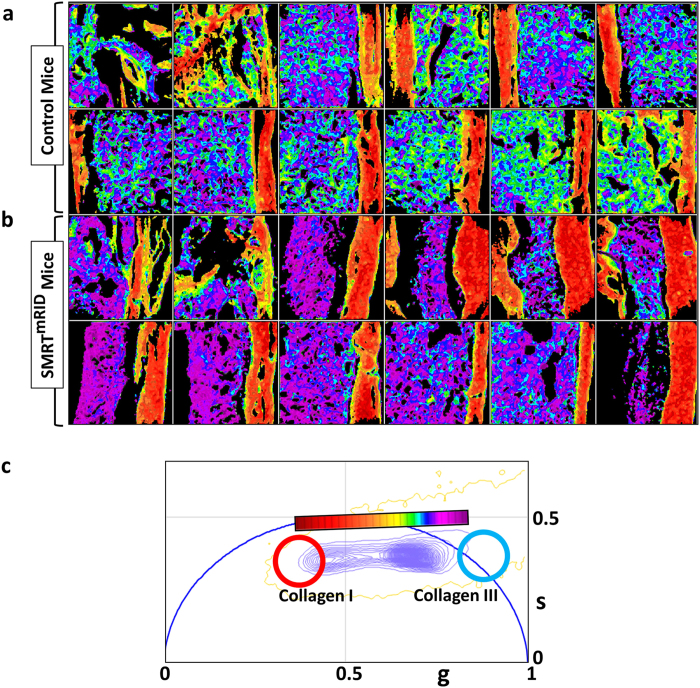
Separation of collagen I and III in normal and fibrotic bone marrows. (Fig. 5a) and (Fig. 5b) show the phasor masked FLIM images of the non-fibrotic wild type mice and fibrotic SMRT^mRID^ mice, respectively. The more violet color in the Fig. 5b is representative of higher contribution from collagen III. Figure 5c shows the phasor plot and the continuous cursor used for the analysis.

The femur slices from two different mice; one wild type control mouse and one from SMRT^mRID^ mouse were imaged using the phasor approach to the fluorescent lifetime imaging. The different areas of these two samples imaged using the FLIM technique are shown in Supplementary Figure S3. These images were then analyzed using the continuous cursor analysis in the phasor plot. As mentioned in materials and methods section, one of the key unique features of the phasor approach is that, in this approach a continuous color scheme can be used to show differential contribution of two separate species in any individual pixel. Figure 5c shows the continuous color scheme used to show the differential contribution of collagen I and collagen III in these images. The more red color is representative of the more collagen I rich areas and the more violet color is representative of more collagen III rich areas. A comparison between the phasor masked images of the wild type mice in Fig. 5a and the SMRT^mRID^ mouse femurs slices in Fig. 5b shows that while the periphery in both cases is made of mostly collagen I, the bone marrow of the SMRT^mRID^ mouse is more violet in color and hence have more contribution from collagen III. This is similar to the results of staining shown before^32^. Thus Fig. 5 demonstrates that phasor approach to FLIM can indeed be used image fibrosis in tissues.

## Materials and Methods

### Preparation of collagen gels

Collagen I from rat tail (Cat. No. – 354236) was bought from Corning Incorporated (Tewksbury, MA). Collagen II from chicken sternal cartilage (Cat. No. – C9301-5MG) and, collagen III (Cat. No. – C4407-1MG), collagen IV (Cat. No. – C7521-5MG) and Collagen V (Cat. No. – C3657-1MG) from human placenta were purchased from Sigma Aldrich (St. Louis, MO). All the collagens gels were prepared using the following procedure. The collagen samples were first diluted to 3.75 mg/ml. The eight chamber borosilicate coverglass system (Lab-Tek) was placed on the refrigerator at 4 °C. All the components were placed on ice to decrease the temperature shock. In a 2 ml sterile tube 317 μl water and 533 μl collagen was added and vortexed to ensure complete mixing. 100 μl 10X PBS pre-mixed with phenol red was added to this solution while vortexing and then neutralized with 0.5 N NaOH very slowly until the appearance of light pink color. 350 μL of this collagen mixture and 50 μL of 1X PBS were added to the wells of the Lab-Tek chamber. The chamber was placed at 20 °C for one hour and then transferred to the 37 °C incubator overnight and then imaged the next day. The gel containing the mixture of the collagen I and III was prepared by mixing 270 μL of collagen I and 90 μL of collagen III prior to the addition to Lab-Tek chambers.

### Biological sample preparation

The femurs of both wild type mice and SMRT^mRID^ mice were simultaneously decalcified and fixed with CAL-EXII (Fisher Scientific, USA). Then femurs were embedded and frozen in O.C.T. compound (TissueTek, USA). 10 mm frozen sections were obtained using Leica CM 1850 Cryostat (Leica, Germany). SHG and FLIM images were obtained for these bone slices. Generation and initial characterization of SMRTmRID mice are described previously^34^. These mice were further backcrossed for 4 more generations to sv129. Only age matched male mice (average cohort size 6–10) were randomly assigned and used. All mice were bred and maintained in the Salk Institute animal facility under specific pathogen free conditions. Procedures involving animals were reviewed and approved by the Institutional Animal Care and Use Committee (IACUC) at the Salk Institute, and conformed to regulatory and ethical standards. The methods were carried out in accordance with the approved guidelines. The mouse studies were not blinded, as the same investigators performed the grouping, dosing and analyses, rendering blinding of the studies unfeasible.

### Microscopy

The fluorescence lifetime imaging and the second harmonic generation imaging were carried out using the homebuilt DIVER (*Deep* Imaging via Enhanced-Photon Recovery) microscope. The details of this microscope construction are explained elsewhere^35^,^36^,^37^. Briefly, the DIVER microscope is based on an upright laser scanning fluorescence microscope. The main difference from a regular upright microscope is on the emission path of the instrument. Here the sample is placed directly on top of the filter wheel assembly and the filter wheel is placed right on top of a wide area PMT. The collagen gel samples were excited with 710 nm line of a Deep See MaiTai laser with a 40X water immersion objective (Olympus Plan Apo). The bone samples were excited with a 20X air objective (Olympus). Different filter sets were used to select either the SHG or the fluorescence generated in the samples. A combination of UG11 and BG39 (used to protect the PMT from direct excitation) creates a window of 350 nm ± 20 nm (FWHM) and was used to collect the SHG signal. Another filter with a window of 400 nm to 560 nm was used for the collection of the fluorescence signals. The signals were recorded using the FLIMBOX and directly transferred to the phasor plot^38^. The second harmonic signal has a lifetime of zero and appears at the position of s = 0 and g = 1 at the phasor plot. Fluorescence signals have non-zero lifetime and appear elsewhere in the phasor plot. A solution of Rh110, having a lifetime of 4 ns, was used for the calibration of the phasor and used as the standard for all the samples.

### Phasor approach to fluorescence lifetime^26^,^29^

The lifetime signals originating from the different collagens samples were analyzed by the phasor approach to fluorescence lifetime. The details of this approach for both the TCSPC (time correlated single photon counting) and phase and modulation measurements are explained elsewhere and have been used extensively in biological samples^14^,^28^,^29^,^31^,^38^. Briefly, the intensity decay originating from each point of an image is transferred to the phasor plot and creates a single point. A particular population in the phasor plot can then be chosen using a colored cursor and the fluorescence intensity image can be painted accordingly. This results in a fit free method to analyze FLIM images. Different populations corresponding to different lifetimes can easily be selected in the phasor plot and thus the intensity image can be masked according to the fluorescence lifetime. This is instantaneous and unlike the TCSPC approach, does not require a multi-exponential fitting at every pixel of an image. Thus the phasor approach is computationally much less expensive and faster. If the intensity decay at any pixel can be defined by a mono-exponential, then the phasor point originating from that pixel appears in the semicircle shown in blue in the phasor plots (called the universal semicircle). Multi-exponential decays result in phasor points inside the universal semicircle. A mathematical property of this method is that if at one pixel, there are contributions from two or more different exponential components, i.e. different phasor positions on the universal circle, then the corresponding point in the phasor plot of that pixel lies along the line or lines joining the those individual components at the universal circle. This is called the law of linear combination. According to this law, the relative contribution of those components can be obtained graphically by calculating the distance between the combination point in the phasor plot and the individual component positions in the universal circle. The SHG signals have a lifetime of zero as the signal from the SHG is coherent with the laser and they appear at s = 0, g = 1 in the phasor plot. Fluorescence signals from the collagens have non-zero lifetime and appear inside the semicircle.

## Additional Information

**How to cite this article**: Ranjit, S. *et al.* Imaging Fibrosis and Separating Collagens using Second Harmonic Generation and Phasor Approach to Fluorescence Lifetime Imaging. *Sci. Rep.* **5**, 13378; doi: 10.1038/srep13378 (2015).

## Supplementary Material

Supplementary Information
