# Supplementary material for: Imaging Fibrosis and Separating Collagens using Second Harmonic Generation and Phasor Approach to Fluorescence Lifetime Imaging
Source: Sci Rep. 2015 Aug 21;5:13378. doi: 10.1038/srep13378 (PMC4543938; doi:10.1038/srep13378)
Supplement: Supplementary Information [file srep13378-s1.pdf]

Supplementary Information

Imaging Fibrosis and Separating Collagens using Second Harmonic Generation and Phasor Approach to Fluorescence Lifetime Imaging

Suman Ranjit[1], Alexander Dvornikov[1], Milka Stakic[1], Suk-Hyun Hong, Moshe Levi[2] , Ronald M. Evans[3] and Enrico Gratton[1*]

[1]Laboratory for Fluorescence Dynamics, Department of Biomedical Engineering, University of California Irvine, California.

[2]Division of Renal Diseases and Hypertension, University of Colorado at Denver, Aurora, Colorado

[3]Gene Expression Laboratory, Salk Institute for Biological Studies, La Jolla, CA

*Corresponding Author: egratton@uci.edu

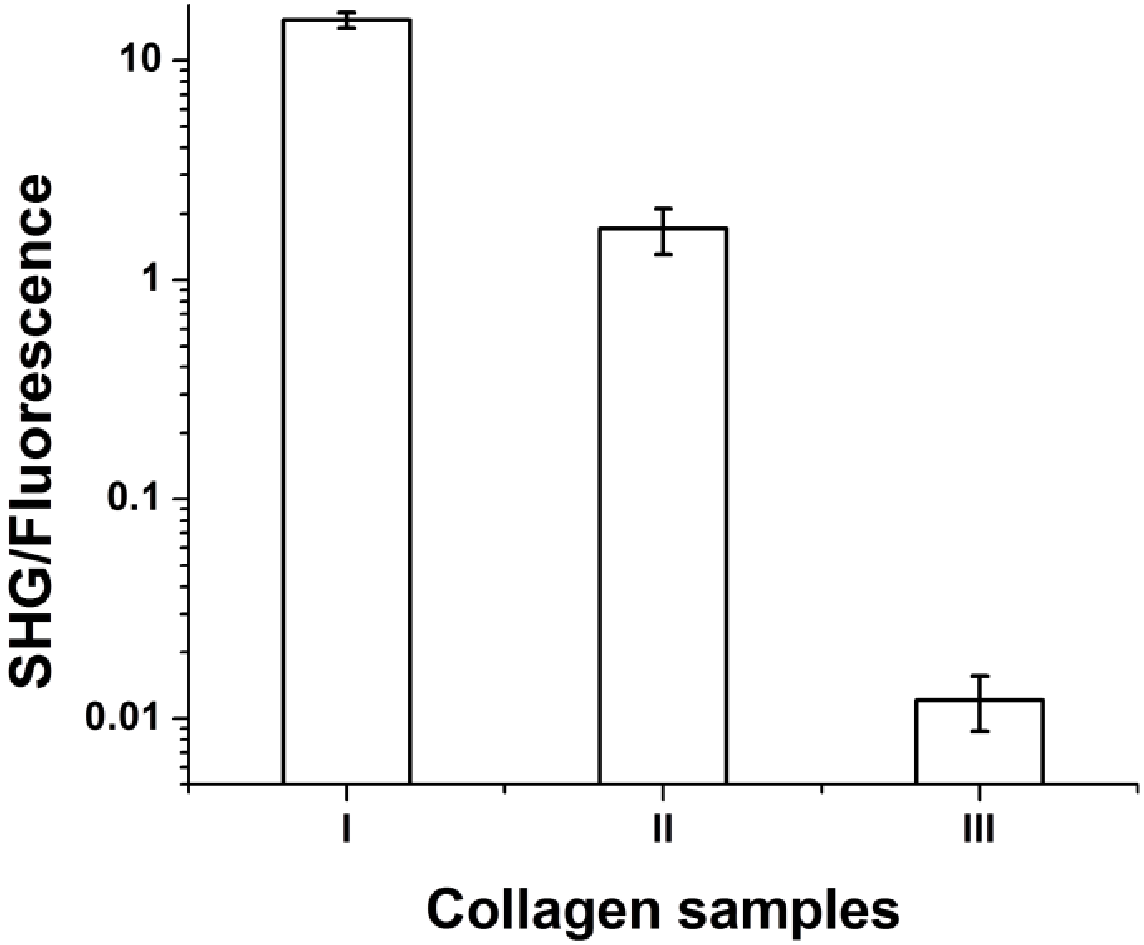

Supplementary Figure 1

Ratio of total intensity in the SHG channel to the fluorescence channel. Collagen I has the highest SHG and lowest fluorescence signal. Collagen III shows the opposite behavior.

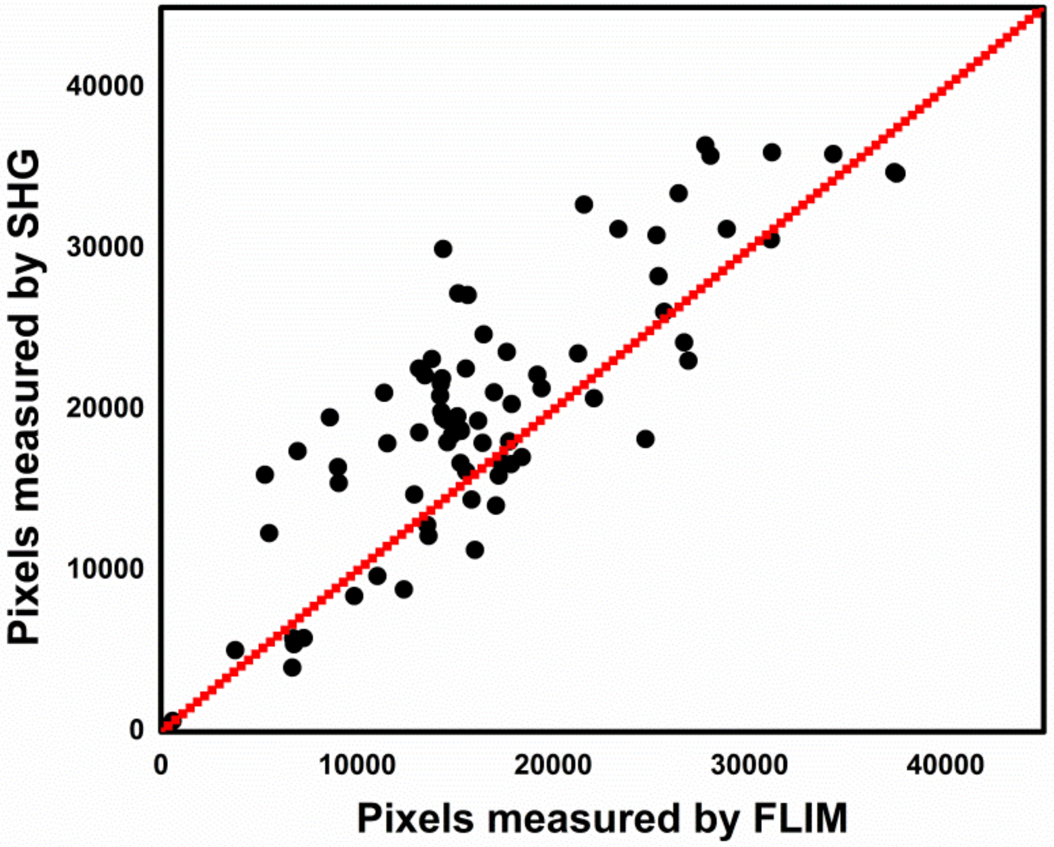

Supplementary Figure 2

Correlation between the number of pixels measured by the SHG and the pixels measured by the red mask (the mask for collagen I) in the FLIM images. In total, 80 total areas were measured for both SHG and FLIM images and the number of pixels in SHG and red masked FLIM images were calculated and plotted for the correlation plot below. The strong correlation between the two types of measurements shows that collagen I can be identified by both SHG and FLIM.

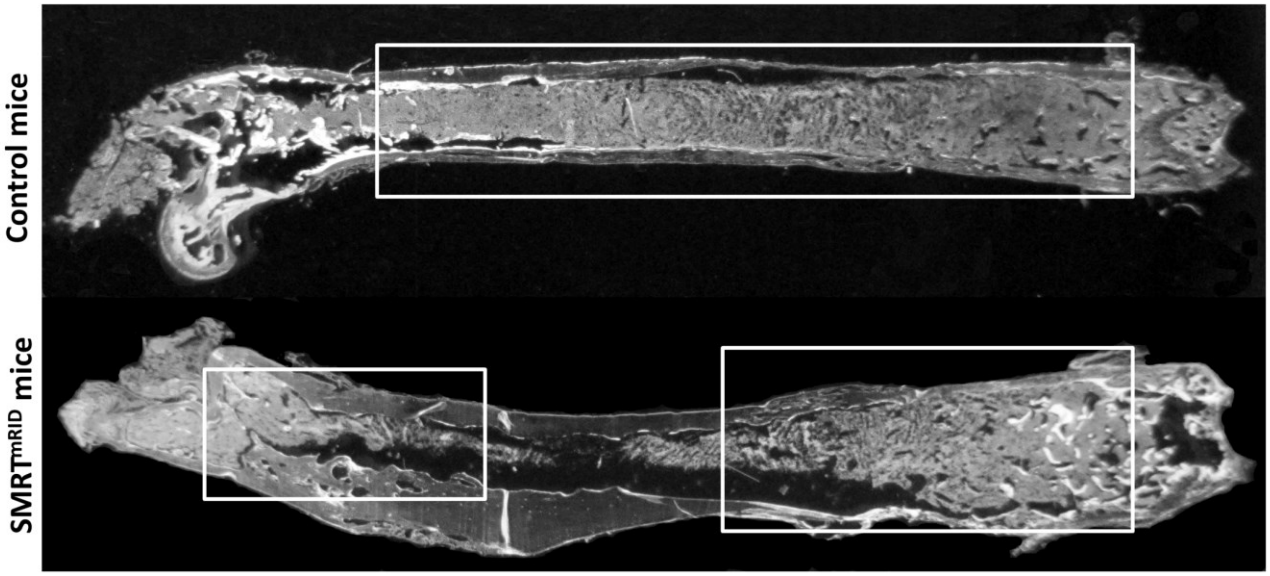

Supplementary Figure 3

Areas of the bones used for the FLIM analysis of Figure 5. The polygon covering the top shows the area measured for the normal WT mouse and the bottom shows the similar for the $SMRT^{mRID}$ mouse.
